# Ornamental Exterior versus Therapeutic Interior of Madagascar Periwinkle (*Catharanthus roseus*): The Two Faces of a Versatile Herb

**DOI:** 10.1155/2015/982412

**Published:** 2015-01-15

**Authors:** Naghmeh Nejat, Alireza Valdiani, David Cahill, Yee-How Tan, Mahmood Maziah, Rambod Abiri

**Affiliations:** ^1^Institute of Tropical Agriculture, Universiti Putra Malaysia (UPM), 43400 Serdang, Selangor DE, Malaysia; ^2^Department of Biochemistry, Faculty of Biotechnology and Biomolecular Sciences, Universiti Putra Malaysia (UPM), 43400 Serdang, Selangor DE, Malaysia; ^3^School of Life and Environmental Sciences, Faculty of Science Engineering & Built Environment, Deakin University, Melbourne, VIC 3220, Australia; ^4^Department of Plant Protection, Faculty of Agriculture, Universiti Putra Malaysia (UPM), 43400 Serdang, Selangor DE, Malaysia; ^5^Institute of Bioscience, Universiti Putra Malaysia (UPM), 43400 Serdang, Selangor DE, Malaysia

## Abstract

*Catharanthus roseus* (L.) known as Madagascar periwinkle (MP) is a legendary medicinal plant mostly because of possessing two invaluable antitumor terpenoid indole alkaloids (TIAs), vincristine and vinblastine. The plant has also high aesthetic value as an evergreen ornamental that yields prolific blooms of splendid colors. The plant possesses yet another unique characteristic as an amiable experimental host for the maintenance of the smallest bacteria found on earth, the phytoplasmas and spiroplasmas, and serves as a model for their study. Botanical information with respect to synonyms, vernacular names, cultivars, floral morphology, and reproduction adds to understanding of the plant while the geography and ecology of periwinkle illustrate the organism's ubiquity. Good agronomic practices ensure generous propagation of healthy plants that serve as a source of bioactive compounds and multitudinous horticultural applications. The correlation between genetic diversity, variants, and TIA production exists. MP is afflicted with a whole range of diseases that have to be properly managed. The ethnobotanical significance of MP is exemplified by its international usage as a traditional remedy for abundant ailments and not only for cancer. TIAs are present only in micro quantities in the plant and are highly poisonous* per se* rendering a challenge for researchers to increase yield and reduce toxicity.

## 1. Introduction

The genus* Catharanthus* consists of eight species of which seven are endemic to Madagascar and one,* C. pusillus*, is endemic to India.* Catharanthus roseus*, Madagascar periwinkle (hereafter MP), is an important floral species in horticulture and is one of the few pharmacological plants that have a long history. It could be traced to Mesopotamian folklore going as far back as 2600 BCE. This plant still plays a considerable role today in herbal and traditional medicine for treatment of various diseases. The therapeutic properties were ascribed to a number of chemicals in the alkaloid class sometime in the mid-1950s [1, 2].

Medicinal plants have played a key role in world health care [3]. MP apart from being the most important source of natural drugs is also one of the premier model organisms to study plant alkaloid metabolites due to its ability to synthesize a wide range of terpenoid indole alkaloids (TIAs) with a broad pharmaceutical spectrum [4–7]. However, MP is potentially poisonous as are many plants of the dogbane family. Nowadays MP comes in many different varieties and colors ranging from white, hot pink, and mauve to the original pink as a result of breeding experiments. As previously published reviews on MP mostly concentrate on the pharmaceutical and chemical compounds of the herb, lack of information about the other features of the species is extremely tangible. The aim of this review is to provide insights into agrotechnological, biological, ecological, and medicinal aspects (particularly anticancer compounds) of MP as well as updated information on the new cultivars that have been introduced to the horticultural industry.

## 2. Botany

### 2.1. Taxonomic Classification

Inconsistency of nomenclature in the literature is due to changes made in plant taxonomic classification with continuous research [8]. In the appellation of* Catharanthus roseus* (L.) G. Don,* Catharanthus* means clean or pure flower in Greek,* roseus* signifies being rose-colored in Latin, L. is the standard abbreviation for Linnaeus, who first published the plant's description, and G. Don refers to George Don, who named the flower as such in 1837 [6].

*Catharanthus roseus* known as the Madagascar periwinkle (MP) was formerly classified as the species* Vinca rosea* L. (1759) (basionym or original name),* Pervinca rosea* (L.) Moench (1794),* Lochnera rosea* (L.) Rchb. ex Endl. (1838), and* Ammocallis rosea* (L.) Small (1903), which are homotypic synonyms [9].* Vinca guilelmi-waldemarii* Klotzsch is recognized as a heterotypic synonym [10].

Below describes the classification of the* Catharanthus roseus* species.

 Domain: Eukarya: eukaryotes.
 Kingdom: Plantae: plants.
 Subkingdom: Tracheobionta: vascular plants.
 Superdivision: Spermatophyta: seed plants.
 Division: Magnoliophyta: flowering plants.
 Class: Magnoliopsida: dicotyledons.
 Subclass: Asteridae.
 Superorder: Gentiananae.
 Order: Gentianales.
 Family: Apocynaceae: dogbane.
 Subfamily: Rauvolfioideae.
 Tribe: Vinceae.
 Genus:* Catharanthus* G. Don.
 Specific epithet:* roseus* (Linnaeus) G. Don.
 Botanical name:* Catharanthus roseus* (Linnaeus) G. Don (1837): Madagascar periwinkle [8, 11–13].

Vernacular names of the herb in various cultures and languages have been listed in Table 1.

### 2.2. Morphological Characteristics

**Figure 1:**
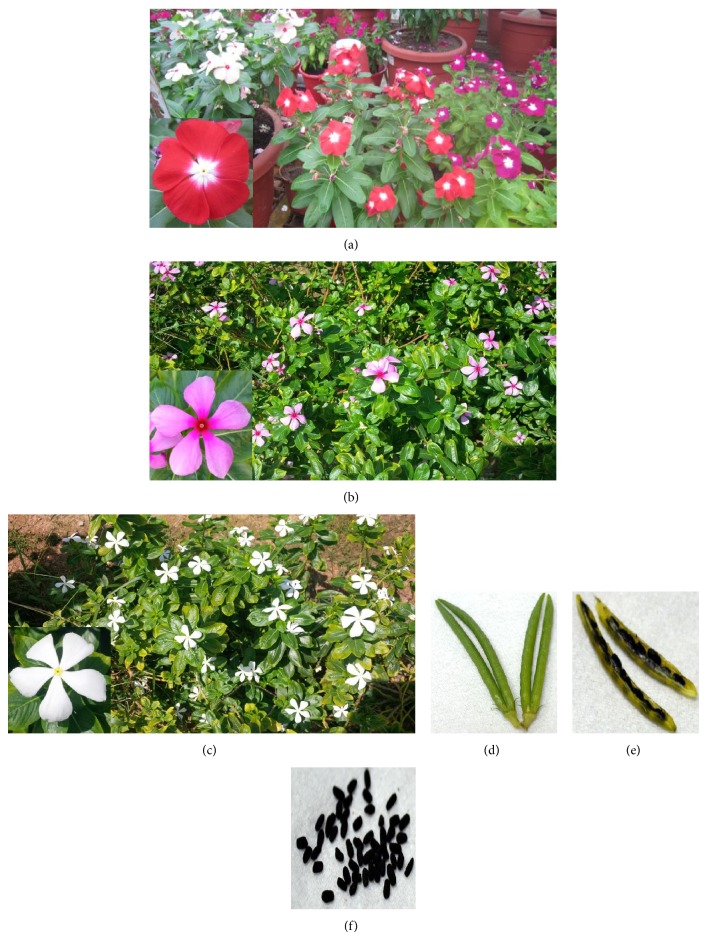
(a) Artificial hybrids of MP in flower stage; (b) wild types with purple flowers “rosea”; (c) wild types with white flowers “alba”; (d) fruits which are composed of two free narrow cylindrical follicles; (e) seeds inside the follicle; (f) seeds. All images represent the real sizes, approximately. The artificial hybrids are clearly differentiated from those wild types due to their floral architecture and petals structure which are wider, thicker, and more compressed than the wild ones.

MP is a perennial or annual evergreen, semishrub or herbaceous plant that grows up to one meter in height and secretes milky latex. The roots extend to 70 cm in depth. The leaves of the plant are elliptical to oblong in shape, 2.5–9 cm long, 1–3.5 cm broad, glossy green above, and pale green below with a pale midrib and opposite in orientation. Petioles are green or red and 1–1.8 cm long. The inflorescence is racemose. Flowers are pentamerous, actinomorphic, and colored pink, rose-purple, blue, salmon, scarlet, or white with purple, red, pink, pale yellow, or white “eye” in the center and a mauve throat (Figures 1(a)–1(c)). The corolla tube is cylindrical, 2-3 cm long with five petal-like lobes. Stamens are inserted 0.4–0.6 cm below the corolla mouth, consisting of very short white colored filaments and filiform, subsessile anthers. Pistils range from 17 to 26 mm in length, made up of two narrow, long carpels consisting of a glabrous stigma, style, ovary, and 2-seriate ovules. Each fruit is composed of two free, narrow cylindrical follicles, 2–4.5 cm long and 3 mm wide, which houses 10–20 oblong, minute seeds, 2-3 mm in length (Figures 1(d)–1(f)). These seeds comprise black cotyledons which are flat and slightly shorter than the radical and a scanty endosperm [9, 14–16].

## 3. Geographical Distribution and Ecology

MP is a plant species native to Western Indian Ocean's large island of Madagascar next to Africa. It has been introduced as a popular ornamental plant in many tropical and subtropical regions worldwide. This herb is widely cultivated commercially in Spain, United States, China, Africa, Australia, India, and Southern Europe for its medicinal uses. The drugs derived from this plant find major markets in USA, Hungary, West Germany, Italy, The Netherlands, and UK [17–19].

MP is a tolerant plant against abiotic stresses such as drought and salinity, which can survive in a variety of habitats such as sandy soils, shrublands, grasslands, inland river banks, dunes in savannas, dry wastelands, houses, roadsides, and even beaches and limestone rocks all due to its hardiness. It can be found proliferating at a range of altitudes from 0 to 900 meters. MP prefers a soil pH of 5.5–6.5, can withstand salt up to 2000 ppm, and has excellent heat and drought tolerance. It thrives well in dry, frost-free, and humid environments with favorable moisture conditions, under full sun or partial shade, and in soils that are well-drained. It bears flowers and fruits the whole year in warm climates. MP cannot withstand too much water, wet soils, or a cool spring. Under adverse weather or in poorly drained soils, MP turns yellow-green while overwatering could lead to bacterial and fungal rot diseases of stem and root [9, 10, 20].

## 4. Genetics

### 4.1. Cytology

The chromosome number of all species of the genus* Catharanthus* is 2*n* = 16 [9, 21, 22] with a genome size of 1500 Mbp (van Iren, unpublished). Doubling of chromosome number, tetraploidy, has been induced by colchicine treatment that resulted in an increase in TIAs, larger stomata, branches, and leaves, although there was reduced pollen fertility and poor seed set compared with diploid plants [23, 24].

### 4.2. Reproduction

MP is a unique species because of its self-compatibility unlike most of the other species in the family. However, intraflower self-pollination does not normally occur in periwinkle because of the physical separation between the stigma and anthers, a phenomenon known as reverse herkogamy, when the stigma is recessed below the level of anthers [25–28]. Even so, some periwinkles contain elongated ovaries or styles, thus allowing for intraflower self-pollination. Basically, MP is an allogamous (cross-pollinating) species. The degree of outcrossing varies with environmental conditions and the presence of seasonal pollinators, mainly butterflies and moths, whereby the floral structure is adapted to pollination by these long-tongued insects [29]. Self-incompatible strains of MP engage in natural interspecific hybridization which is found to be common locally in Madagascar [18, 30]. But in all reality, both types of pollination occur in MP.

### 4.3. Genetic Diversity

Although many aspects of alkaloid biosynthesis have been investigated, the genetic variation between accessions in relation to alkaloid content and the effects of breeding for flower color or growth habit on the levels of vinblastine (VBL) and vincristine (VCR) are still poorly understood. Dissimilar cultivars accumulate TIAs in various parts of the plant. Hence, efforts need to be made to identify accessions out of the vast resources of naturally occurring MP germplasm for their chemotherapeutic potential, determine the type of plant tissues accumulating these active compounds, and administer genetic improvements for higher yield of alkaloids [31, 32]. A summary of genetic relatedness among* C. roseus* accessions based on different markers is shown in Table 2.

## 5. Agrotechnology

### 5.1. Cultivation

Even though many investigations have been carried out over the last few decades on the phytochemical and therapeutic properties of MP, few studies have been conducted on the agronomic and genetic aspects of this herb. MP is very easy to cultivate. The seed germinates within a week in the dark. Seeds are easily sown directly or seedlings transplanted at a seeding rate of 2-3 kg/ha and 0.5 kg/ha, respectively [33–35]. In a temperate climate, seeding takes place from March to April, the best temperature for seed germination being 25–30°C. Seeds should be planted with a spacing of 45 × 30 cm or 45 × 45 cm. Transplanting takes place between June and July or September and October in the case of 45–60-day-old seedlings spaced at 45 × 20 cm. Softwood stem cuttings or apical semiripe cuttings could also easily be grown in spring or summer under lighted conditions at 20°C and on drained compost [19, 36].

Effects of different levels of nitrogen application rates from 50 to 150 kg/ha on plant yield have been tested with 150 kg/ha giving the best response [37–41]. Root and shoot dry weights were greatest when high nitrate-N to ammonium-N ratio fertilizers were employed with high levels of ammonium-N having an adverse effect [20].

A planting density of 75000 plants/h and nutrient dose comprising a mixture of 15 t/ha farm yard manure (FYM) plus 80 kg/ha N with irrigation (4-5 times) or 15 t/ha FYM + 40 kg/ha N under rainfed conditions are recommended regimes in India. Dry yield (DY) was reported at 1.8 t/ha for leaves and 0.8 t/ha for roots [42]. 3.06 t/ha DY could be obtained with N, P (P_2_O_5_), and K (K_2_O) application at a rate of 150 : 40 : 40 kg/ha [40]. Mineral nutrition can increase the yield and the alkaloid content in periwinkle [43]. Nitrogen fertilization has been found to increase leaf and root yields, significantly. Alkaloids are nitrogenous compounds; therefore, nitrogen may play an important role in the biosynthesis and accumulation of alkaloids in plants [40].

Planting distance 30 × 20 cm produced the highest foliage and root yields [44]. Highest foliage yield was also reported at 45 × 20 cm distance with an NPK 20 : 30 : 30 kg/ha formulation [45]. An early June planting, spacing at 30 × 50 cm, NPK application of 150 : 40 : 30 kg/ha, and a late September harvest are recommended for higher yields in Iran [41]. The best sowing date under temperate climatic conditions in Poland is the second half of May, an optimum planting distance is 60 × 60 cm, while harvesting in early September is ideal [19, 46].

### 5.2. Wild Types and Breeding Synthetic Cultivars

There are two types of MP in the wild, one with pink flowers and reddish stems and the other white flowers and green stems often found growing sympatrically. MP has been bred since the 1920s [47]. Over the past two decades, conventional breeding techniques conducted by horticulturists and seed companies entailed crossing* C. roseus* with other species that resulted in a large number of marvelous new cultivars or varieties. Breeding not only improved floral traits such increasing color range, blooms, and size; enhancing plant growth; augmenting tolerance to disease and cooler growing conditions, but also boosted herbage and alkaloid yield. For example, Dhawal white flower cultivar not only produces a higher plant and alkaloid yield, but also is dieback-tolerant [48]. Nirmal white flower cultivar can endure salt and fungal infection by* Pythium aphanidermatum* and* Phytophthora nicotianae* [15] and possesses a coveted leafless inflorescence (lli) architecture that increased the flower frequency [49].

The different cultivars are usually arranged in series, and subsequent grouping is based on corolla color [50]. Periwinkle plants now are readily available for gardeners worldwide in pink, deep rose, red, scarlet, white, white with a red eye, lavender blue, peach, apricot, orchid, burgundy, and many other shades. An international register of* Catharanthus* cultivars was published in 1998. There are few reliable compendia on this topic, and reliance has to be placed on information from the web. Series and cultivars of MP [10, 47, 51–54] are presented in Tables 3 and 4, respectively. There are also nonseries selections that include Apricot Delight, Blue Pearl, Parasol, Morning Mist, Merry-Go-Round, Fanfare Orchid, Dwarf Little Salmon [55], Pink Panther [56], Santa Fe [57], and Snow Flake.

There have been numerous studies performed on two varieties, rosea and alba, of MP. All the results revealed that rosea is superior to alba in the overall performance of the plant. Rosea has higher alkaloid content in leaves and roots and more antioxidant activity than alba; rosea fared much better under water stress [58–60]. Triadimefon treatment increased the antioxidant potential and alkaloid ajmalicine content in rosea more than in alba [61].

### 5.3. Horticulture

MP is an easy growing and spreading perennial herb that has multitudinous horticultural uses: decoration, annual ground cover, bedding, edging, border, container gardening, mass planting, naturalizing, hanging basket, and wall cascade. Surprisingly, the herb is naturalised for medicinal purposes in many parts of the world while it is considered as a weed or as an ornamental plant in the other parts. This conflict is the result of the MP's long history as a horticultural variety, especially over the past two centuries [62]. Horticultural practices caused changes in the distribution and biology of MP and this, in turn, generated changes in people's valuation of the species [62]. The new lines are novel in displaying an increased number of flowers on the inflorescence unhindered by leaves. In the new floricultural lines the overflowering “LLI” trait was combined with variation in plant height, petal and eye colors, and tolerance to the common fungal diseases [60].

### 5.4. Model Plant in Phytopathology

MP acts as a unique host for a series of microorganisms such as bacteria and fungi. Hence, the herb serves as a model plant for studying the biology of microbial pathogenesis. As an invaluable model indicator plant for mollicutes in plant pathology, MP has no peers. It is an excellent experimental host for most phytoplasmas and spiroplasmas where high titres are achieved [63]. MP is highly susceptible to phytoplasma infection from different crops and exhibits highly distinctive symptoms such as phyllody and virescence. This popular herb is commonly used as a source plant to maintain mollicutes by grafting, dodder, or vector transmission. Infecting mollicutes induce symptoms on this experimental host similar to those on the original hosts [64]. MP has also been used as a model plant to study the interaction between the endophytes* Methylobacterium mesophilicum* and* Xylella fastidiosa* [65].

## 6. Diseases and Management

### 6.1. Diseases

Periwinkle plants are known to be susceptible to the aster yellows (16SrI) group phytoplasma in Argentina [66], Egypt [67], India [68], Malaysia [69, 70], and Myanmar [71] and have also been found to be naturally infected with spirea stunt (16SrIII-E), peach yellow leaf roll (16SrIII-A), clover proliferation (16SrVI), potato witches' broom (16SrVI-A) [72], Mexican periwinkle virescence (16SrXIII) [73], and Malaysian periwinkle virescence (16SrXXXII) [74, 75]. MP was the first nonrutaceous plant found naturally infected with* Spiroplasma citri* in the United States [76]. This pathogen was subsequently discovered in Mediterranean countries, including Morocco, Syria, Cyprus, and Turkey [77], and in Southeast Asia, Malaysia [78]. Periwinkle is also known to be susceptible to the cucumber mosaic virus (CMV), and disease incidence has been reported in Australia [79], India [80], and Malaysia [81].

Bedding plants such as periwinkle are susceptible to many damping-off diseases. MP is prone to blight, canker, leaf spot, and root rot. Pathogenic fungi include* Alternaria* (leaf spot),* Rhizoctonia solani* (stem, crown, and root rot), and* Phytophthora parasitica* Dast. (foliars and stems).* P. parasitica*, a soilborne fungus, causes serious losses and death in the periwinkle with reports from India and the United States [82–85].* Fusarium* root rot has been reported from Taiwan [86]. Blight caused by* P. nicotianae* (syn. =* P. parasitica*) is one of the most damaging diseases of MP in Florida [87]. Other known blights include twig blight caused by* Colletotrichum dematium* [88], foliar blight brought about by* P. tropicalis* in Virginia [89], and gray mold blight affected by* Botrytis cinerea* in the United States, Italy, and Taiwan [90–92]. Other fungi identified to incite disease in periwinkle seedlings in Taiwan include* Botrytis cinerea*,* Colletotrichum gloeosporioides*,* Fusarium oxysporum*,* F. solani*,* P. parasitica*,* Pythium aphanidermatum*,* Rhizoctonia solani*,* Sclerotinia sclerotiorum*, and* Sclerotium rolfsii* [92]. Black root rot through* Thielaviopsis* infection is the most serious root disease in MP because it is very difficult to control [47]. Periwinkle rust due to* Puccinia vincae* attack (http://donsgarden.co.uk/) is another disease to be noted. Fungal problems can occur under humid or wet conditions so care has to be exercised to prevent overwatering. Insect pests are no major concern, although aphids, thrips, spider mites, mealy bugs, and scale insects can infest this plant [47].

### 6.2. Disease Management

Environmentally, a full-sun planting location reduced humidity levels, and increased air circulation around the plants should be targeted. Potting mixes have to be pathogen-free and new containers prepared for each planting. Sanitation-wise, weeds should be kept down, infected soils and plants removed immediately upon detection, all equipment kept clean, and the planting area cleared of old potting media and plant debris. In fertilization and irrigation, avoid heavy fertilization and overwatering of plants. Overhead irrigation and prolonged periods of leaf wetness should be avoided with watering done only during the day. Soil fertility has to be properly adjusted to pH maintained at 5.5. Transplants need to be spaced at least 10–12 inches apart. Biocontrol of soilborne fungal pathogens could be attempted with* Trichoderma virens*, binucleate* Rhizoctonia*,* or Burkholderia cepacia* [47, 87, 93–96].

## 7. Phytochemistry

**Figure 2:**
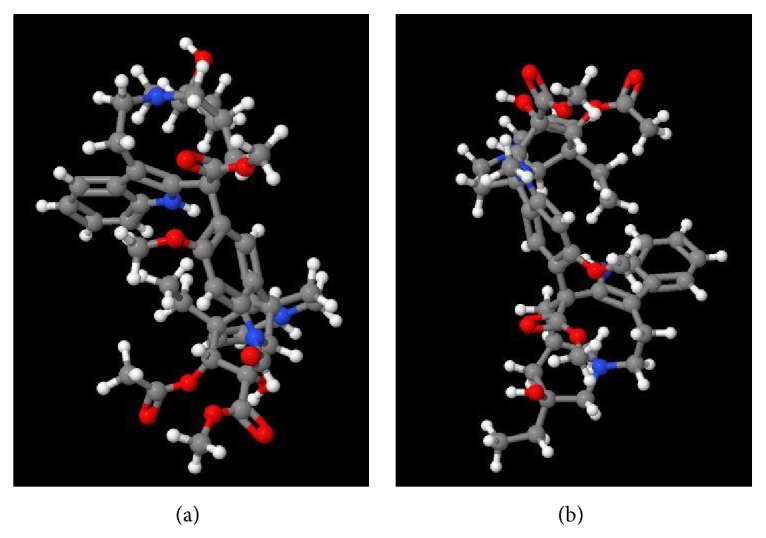
The three-dimensional structures of the main anticancer compounds of MP, (a) vincristine and (b) vinblastine, in a ball-stick model (http://www.chemspider.com/ImageView).

Two commercially and pharmacologically important cytotoxic dimeric alkaloids of MP, vinblastine and vincristine, have been widely used for cancer chemotherapy, which are only present in extremely low yields in the leaves [31]. The three-dimensional structure of these two compounds has been featured in Figures 2(a)-2(b). In addition to alkaloids and phenolics (as the most important compounds of MP), the presence of different chemical groups such as polyphenols, alkaloids, steroids, flavonoid glycosides, anthocyanins, and iridoid glucosides has been confirmed in different parts of MP [97]. However, some evidence implicates the availability of similar compounds in the leaves and stems of the plant, but the same does not comply with the contents of seeds and petals [98]. Flower extract of MP has been used as a natural acid-base indicator [99, 100].

### 7.1. Major Alkaloids

All parts of the plant contain several active alkaloids with an indole moiety. More than 130 indole alkaloids, collectively termed terpenoid indole alkaloids (TIAs), have been extracted from periwinkle [31, 101–104]. Some of these alkaloid compounds have distinct medicinal properties. The alkaloid content is highest at the flowering stage [50]. The principal alkaloids present in the aerial (nonfloral) parts are VBL (vincaleukoblastine, VLB), VCR (leurocristine, vincaleurocristine), vincarodine, vincoline, leurocolombine, viramidine, vincathicine, vincubine, isositsirikine, vincolidine, lochrovicine, catharanthine, vindoline, leurosine, lochnerine, tetrahydroalstonine, and vindolinine. Ajmalicine (raubasine), serpentine, and reserpine are the main alkaloids in the root while coronaridine, 11-methoxy tabersonine, tetrahydroalstonine, ajmalicine, vindorosidine, and vincristine dominate in the flower. However of the over hundred alkaloids discovered, only five consisting of vinblastine, vincristine, 3′,4′-anhydrovinblastine, serpentine, and ajmalicine are marketed [102]. Other* Catharanthus* species such as* C. longifolius*,* C. trichophyllus*, and* C. lanceus* are known to possess vindoline type alkaloids.

### 7.2. Major Phenolics

Phenolic compounds are a group of metabolites available in all plant species. These compounds can range from simple compounds bearing just one phenolic hydroxyl to some more complex ones, like flavonoids, which are often polyphenols. Besides alkaloids, MP produces a wide spectrum of phenolic compounds with radical scavenging ability, including C6C1 compounds such as 2,3-dihydroxybenzoic acid, as well as phenylpropanoids such as cinnamic acid derivatives, flavonoids, and anthocyanins [97]. Mustafa and Verpoorte [97] have listed the most important phenolic compounds of MP, including 2,3-DHBAG, SA; SAG, benzoic acid, 2,5-DHBA, and 2,5-DHBAG, gallic acid, glucovanillin, vanillic acid, glucovanillic acid, vanillyl alcohol, vanillyl alcohol-phenyl-glucoside, C6C3/conjugated C6C3:* trans*-cinnamic acid, hydroxytyrosol, ferulic acid, chlorogenic acid, C6C3C6/conjugated C6C3C6: kaempferol, trisaccharides, quercetin, syringetin glycosides, malvidin, malvidin 3-O-glycosides, malvidin 3-O-(6-O-p-coumaroyl), petunidin, petunidin 3-O-glucosides, and petunidin 3-O-(6-O-p-coumaroyl).

## 8. Phytobioactivity

### 8.1. Ethnobotanical Importance

Decoctions of MP are mentioned in folklore remedies for treatment of diabetes, malaria [105], dengue fever, dysentery [106], insect bites [107], skin infection, diarrhea, leukemia, eye irritation, dyspepsia, dysentery, toothache, sore throat, and lung congestion [106]. The root of the plant is reported to be a tonic and possess hypotensive, sedative, and tranquillizing properties [108]. In Ayurveda, it is used for treating diabetes. Hypoglycemic activity of aqueous extracts from MP has been proved in modern studies, as well [109]. In Madagascar, the bitter and astringent leaves have been applied as an emetic; roots have been used as a purgative, vermifuge, depurative, hemostatic agent and toothache remedy. In the Philippines, the leaf decoction is an herbal treatment for diabetes, young leaves are for stomach cramps, and root decoction is for intestinal parasitism. Mauritians employ infusion of leaves for indigestion and dyspepsia. In India (Orissa and Assam), juices from the leaves are used to treat wasp stings while roots and leaves are utilized as anticarcinogenic agents [18, 110].

MP floral extracts enjoy wide usage in many countries as a remedy for many ailments: Cuba and Jamaica: eyewash for infants; Bahamas: asthma; Bermuda: high blood pressure; Indo-China: dysmenorrhea; Surinam and throughout the Caribbean: eye irritation/infections, malaria, menstrual pains, and diaphoresis. Ugandans put faith in leaf infusions to treat stomach ulcers while Batswana ground leaves in milk for mature abscesses. In Togo, a root decoction is taken to treat dysmenorrhoea [18, 110]. Even in developed countries such as Europe, MP is used as a folk remedy for diabetes for centuries. In Hawaii, the plant is boiled to make a poultice stop bleeding. The Chinese have widely applied this versatile herb in the treatment of leukemia, hypertension, lymphosarcoma, and giant follicular lymphoma while adapting it as an astringent, diuretic, and cough remedy. Similarly, in central and south America, it is popular as a homemade cold remedy to ease lung congestion, inflammation, and sore throat while it is trusted as an alternative medical treatment source for leukemia in Hong Kong and Korea. Japan has targeted MP for arresting diabetes and malignant lymphatic tumours [111]. In addition, modern research has revealed a broad spectrum of medicinal applications for the herb (Table 5).

### 8.2. Toxicity and Side Effects

MP can be dangerous if consumed orally. It can be hallucinogenic and is cited as such (under its synonym* Vinca rosea*) in the Louisiana State Act 159. TIAs have been applied clinically since the end of the 1950s as major drugs in the treatment of acute lymphoblastic leukemia, non-Hodgkin lymphomas, myeloma, and Hodgkin lymphoma [112]. Despite their benefits, all of the MP alkaloids have neurotoxic activity, especially vincristine, affecting neurotransmission [113]. Vincristine and vinblastine are highly toxic antimitotics, blocking mitosis in metaphase after binding to the microtubules [114]. Moreover, many side effects have been reported for these drugs comprising myelosuppression, alopecia, abdominal cramps, constipation, nausea/vomiting, paralytic ileus, ulcerations of the mouth, hepatocellular damage, kidney impairment, pulmonary fibrosis, urinary retention, amenorrhoea, azoospermia, orthostatic hypotension, and hypertension [113, 115, 116]. The dosage and administration must be carefully controlled to reduce side effects [112].

### 8.3. Formulations and International Trade

The two key pharmaceutical dimeric alkaloid compounds, VBL and VCR, exist mainly in the aerial parts of the plant in extremely low concentrations, the latter quantitatively much less than the former [50, 117–119]. Vincristine sulfate, originally known as leurocristine, is the only effective antileukemic drug that reduces white blood cell count drastically; since the 1950s, it has increased the survival rate of children with leukemia from 20% to 80%. It is one of the most expensive plant products on the market with considerable side effects. Vinblastine similarly decreases the quantity of white blood cells in the blood [112]. Vinblastine sulfate has now been marketed for more than 40 years as an anticancer drug. It has proven effective against Hodgkin's disease. Vincristine sulfate and vinblastine sulfate are being sold for a total US$ 100 million per year [50, 120]. Developing food stuff incorporated by fresh leaves of MP gives rise to the economic importance of the herb while such products possess both pharmaceutical and nutritional properties, simultaneously [121].

These injectable drugs and their semisynthetic analogues such as vinorelbine (VRLB) and vindesine (VDS) interfere with the division of cancer cells [31, 122–126]. Navelbine (5′-noranhydrovinblastine) is a semisynthetic vinca alkaloid with complete microtubule depolymerization ability, broader antitumor activity, and a lower neurotoxicity than VBL and VCR because it selectively interferes with tubulin assembly [127]. Fully synthetic vincristine is far less efficient (only 20% efficiency) compared to the natural product derived from MP, and hence the importance of the species and its bioactive compounds is unchallenged owing to their complex structures [119]. The other valuable therapeutic alkaloid “ajmalicine” is a constituent of hypotensive drugs employed in the treatment of high blood pressure. Two to three hundred tons of MP roots is required for 3600 Kg annual world production of ajmalicine [119].

## 9. Promising Horizons in Biosynthesis of the Phytochemicals

### 9.1. TIAs Pathways Studies

There are two different metabolic pathways for the biosynthesis of terpenoids leading to the formation of the C_5_ central precursor isopentenyl diphosphate (IPP), namely, the classical cytosolic mevalonate pathway and the mevalonate-independent plastidic 2-C-methyl-D-erythritol 4-phosphate (MEP) pathway. The biosynthesis of MP TIAs starts with the condensation of tryptamine (shikimate pathway) and secologanin (MEP pathway) to form the key intermediate strictosidine, the common backbone structure of all TIAs [128]. However, the alkaloid pathway in MP and the involved genes remain partly unknown [128], but gene-to-metabolite networks studies for understanding the biosynthesis of TIAs in MP using the cDNA-amplified fragment length polymorphism (AFLP) markers have revealed valuable information [129]. At the same time, the drawn networks increase the practical potential of metabolic engineering of MP.

### 9.2. Cell and Tissue Culture

VBL and VCR are only produced in very low concentrations in MP. The high therapeutic value and cost of production of the minute amounts of these* bis*-indole alkaloids (VBL and VCR) have prompted extensive efforts to increase their levels by cell-tissue culture and mutation induction [130–132].* In vitro* methods such as cell suspension, hairy root and callus cultures, shoot cultures, metabolic engineering, and regulation studies to improve the phytochemicals production are uncontested shortcut strategies to those time-consuming conventional breeding methods. In other words, when there is a high demand for plant-based pharmaceutics and only low amounts accumulate in plants, plant cell cultures have shown to be an efficient alternative production system of valuable phytochemicals on a large scale. Rapid reproduction and preservation of natural sources are of the beneficial points of such an approach [133]. Plant growth regulators (PGRs) activate plant natural defence mechanisms, thereby leading to increase the biosynthesis of the secondary metabolites in plants. In this regard, application of PGRs like methyl jasmonate (MeJA), dimethyl sulfoxide (DMSO), jasmonates, and salicylic acid as elicitors of secondary metabolites has led to positive results, in recent experiments [128, 134, 135]. Isolation of VBL and VCR using callus culture [136], cell culture [137], shoot culture [138], semisynthetic [139] and totally synthetic approaches [140] is currently considered. Unfortunately, drug supplementation using the aforementioned techniques is limited and cannot meet the existing requirements. Application of artemisinic acid as a useful elicitor has recently been highlighted in suspension-cultured cells of MP [141].

### 9.3. Plant-Fungal Symbiotic Fashion

As mentioned, despite a nonstop explorative trend, the desired level of production of the bioactive compounds such as VBL and VCR has still not been achieved at optimum level [142]. For this reason, isolation of endophytic fungi from the MP plant has been taken into consideration as an alternative solution, more recently [143]. Investigations on the pathobiology of the species have led to very exciting results. Mechanistically, endophytic fungi reside in a symbiotic fashion inside periwinkle plant, imitate their chemistry, and produce the same natural products as their hosts and are thus being screened for the production of the most valuable anticancer compounds of the herb, namely, VBL and VCR. In line with this, researchers took the first steps toward producing VCR using* Fusarium oxysporum*, an endophyte of MP [144, 145]. Later on, Guo and Kunming [146] obtained VBL by* Alternaria* species isolated from the same plant found in China and showed the production based upon thin layer chromatography (TLC) and high performance liquid chromatography (HPLC) only. Eventually, the latest innovation in this area happened by employing the same endophytic fungus (*F. oxysporum*; strain AA-CRL-6), for isolation of both anticancer compounds VBL and VCR in appreciable amounts, successfully [147].

### 9.4. Functional Genomics and Proteomics Exploration

The biosynthesis of VBL includes more than 20 enzymatic phases, in which nine out of 20 are now well characterized at the enzyme and gene level, and several regulatory genes of the pathway (ORCAs) have also been cloned [148]. Proteomic analysis of the plant using two-dimensional electrophoresis led to the identification of 58 proteins, including two isoforms of strictosidine synthase (EC 4.3.3.2), which catalyze the formation of strictosidine in the alkaloid biosynthesis; tryptophan synthase (EC 4.1.1.28), which is required as a supplying source of the alkaloid precursor tryptamine; 12-oxophytodienoate reductase, which is indirectly involved in the alkaloid biosynthesis as it catalyzes the last step in the biosynthesis of the regulator jasmonic acid [149]. Recently, scientists have detected a higher number of proteins using two-dimensional differential in gel electrophoresis (2D-DIGE) technique in two different lines of the herb, 358 proteins in one and 1663 in another line, most of which corresponded to housekeeping proteins or involved in primary metabolism [150]. Of these, a total of 63 enzymes only potentially involved in secondary metabolism, of which 22 were related to TIAs biosynthesis and 16 were predicted transporters putatively involved in secondary metabolite transport. As a matter of fact, these results are important steps towards elucidating the proteome of MP, which are essential to understanding the modality of TIAs biosynthesis [150].

#### 9.4.1. Transformation of Specific Genes

Quantitative analysis of metabolic pathways in MP revealed that metabolically engineered hairy roots can lead to overproduction of TIAs [151]. Recent studies are focused on cloning, characterization, and transformation of the genes involved with the related biosynthetic cycles such as* CrPrx1* that belongs to an evolutionary branch of vacuolar class III peroxidases [148].

#### 9.4.2. Transformation of Genes Affecting the Intermediate Compounds

Recent genetic engineering of MP not only is limited to the genes enhancing the VBL and VCR production directly, but also includes the genes augmenting the biosynthesis of the intermediate compounds such as vindoline, an important intermediate leading to VBL and VCR. Genes such as desacetoxyvindoline-4-hydroxylase-like (*d4h-like*),* ORCA3* (octadecanoid-derivative responsive* Catharanthus* AP2-domain), and* G10H* (geraniol 10-hydroxylase) are suitable examples of the aforementioned trend [152, 153].

#### 9.4.3. Application of Reverse Genetics for Gene Identification

The concept of “reverse genetics” is widely used for characterization of plant enzymes (more precisely genes encoding enzymes involved in alkaloid metabolism) in which the partial sequencing of proteins is carried out after purification of the isolated enzymes from plants or plant cell culture by traditional biochemical chromatography techniques. These sequences are consequently used to identify the corresponding gene from a plant cDNA library [154]. Homology-based cloning of candidate genes and their succeeding functional testing in heterologous expression systems are hastening the pace at which the gene catalogues of alkaloid biosynthesis are developing. For example, putrescine N-methyltransferase, the first enzyme in the nicotine-specific pathway, and an isoflavone reductase-like enzyme putatively involved in nicotine biosynthesis are both expressed specifically in the cortex and endodermis of tobacco root tips. However, intense expression of these enzymes shifts to the xylem parenchyma and outer cortex cells in more differentiated parts of the root [155]. This developmentally regulated spatial expression pattern may be complicatedly associated with nicotine dissemination in tobacco tissues and organs. Tropane alkaloids, as well as nicotine, are mainly synthesized in the root and transported to aerial parts where they accumulate in vacuoles at high levels. Analysis of xylem saps taken from the stem shows that these alkaloids are transported via the xylem [156].

#### 9.4.4. Promoter Engineering

The gene transformation procedure not only is limited to transferring the genes with the “constitutive” promoters, but also can be implemented by fusing the “regulated” promoters to the target gene. The best example of the mentioned process is the agrobacterium-mediated transformation of the hydroxymethylbutenyl 4-diphosphate synthase gene (*HDS*) from the methyl erythritol phosphate (MEP) pathway by replacing an HDS-GUS fused promoter with the constitutive CaMV35S promoter. The functional characterization of the transformed cells confirmed the induction of HDS promoter by several hormonal indicators (auxin, cytokinin, methyl jasmonate, and ethylene) leading to the MIAs production in MP [157].

#### 9.4.5. Gene Silencing

Conceivably, one of the most advanced technologies to scrutinize the MIAs metabolism in MP is the virus-induced gene silencing (VIGS) strategy by establishing* tobacco rattle virus-* (TRV-) based approach [158]. Secologanin is a versatile iridoid and a precursor for the assembly of countless MIAs as well as a number of quinoline alkaloids. In a very recent attempt, employing the VIGS-based method led to the identification of the 7-deoxyloganic acid 7-hydroxylase (*CrDL7H*) gene involved in the third to the last step of secologanin biosynthesis [121]. Silencing the mentioned gene reduced the level of secologanin up to at least 70% and increased the level of 7-deoxyloganic acid to over 4 mg g^−1^ fresh leaf weight compared with the control plants in which this iridoid is not detected. Functional expression of this gene confirmed its priority as a specific substrate for 7-deoxyloganic acid compared to other related substrates. Therefore, it seems that hydroxylation precedes carboxy-O-methylation in the secologanin pathway leading to the MIAs biosynthesis in* C. roseus* [121].

## 10. Conclusion

Nowadays, the demand for natural products and plant-based medicines is growing throughout the world. MP is a remarkable herb owing to its broad spectrum of applications. Apart from its natural supply of anticancer compounds for medicine on the international market, it ranks highly as a popular ornamental plant in the horticultural industry and as a model plant for studies in phytopathology as well as biotechnology. The ultimate goal of current research is to produce MP disease-resistant cultivars with a high content of antitumor alkaloids. Several cultivars have been introduced primarily as ornamental plants with their alkaloid composition, agronomic performance, and genetic affiliations unknown. As such, agronomic and genetic elucidation of new cultivars with exploration of their physiology and secondary metabolism are required for achieving a high quantity and quality output of TIAs. It is unfortunate that MP anticancer drugs have several adverse side effects. This renders further exploration of the clinical pharmacokinetics of MP alkaloids and drug-drug interactions indispensable.

## Figures and Tables

**Table 1:** Vernacular names of Madagascar periwinkle (*Catharanthus roseus*) in different languages^∗^.

Language	Common name
Bengali	Nayantara
Chinese	Chang Chun Hua
Dutch	Roze maagdenpalm
English	Bright-eyes, Cape periwinkle, graveyard plant, Madagascar periwinkle, old-maid, old-maid-flower, rose periwinkle, rosy periwinkle
Ethiopia	Phlox
Creole	Bigalo
French	Pervenche de Madagascar, rose amère, sorcerer's violet
German	Zimmerimmergrün
Creole	Kaka poule
Hindi	Sadabahar, Baramassi, Ainskati, Ushamanjairi
Indonesian	Tapak dara, kembang sari cina
Konkani	Sadapushpa
Malayalam	Nithyakalyani, Savakkottappacha, Ushamalari
Malay	Kemunting cina
Marathi	Sada-phul (Sadaphuli)
Myanmarese	Thin-Baw-MA-Hnyo
Persian	Gul-e-farang
Portuguese	Boa-noite, Boa-tarde, Lavadeira, Pervinca-rosa, Vinca-de-gato, Vinca-de-madagascar, Vina-rósea
Punjabi	Rattanjot
Sanskrit	Nityakalyani
Krio	Ngyange
Afrikaans	Kanniedood
Spanish	Chatas, Chula, Pervinca de Madagascar, Vinca pervinca, Hierba doncella
Swedish	Rosensköna
Tamil	Sudukattu mallikai
Telugu	Billaganneru

^*^References: [9, 17].

**Table 2:** Genetic diversity in *Catharanthus roseus* accessions using different marker systems.

NUA^1^	Type of marker(s)	Clustering method	Similarity indices	Ranges	Genetic variation	References
8	RAPD^2^	UPGMA^7^	NP	86–100	Low-moderate	[159]
8	AFLP^3^	UPGMA	NP	85–100	Low-moderate	[159]
8	^ 1^H NMR^∗^	UPGMA	NP	0–3	Moderate	[159]
8	Chemical (ion content)	UPGMA	Euclidean distance	32–100	High	[160]
14	ISSR^4^	SAHN^8^	Jaccard	0.57–1.00	Moderate	[161]
14	RAPD	SAHN	Jaccard	0.4–0.97	High	[161]
32	Morphochemical/phytochemical	UPGMA	Nei's	55–100	Moderate	[131]
50	Phytochemical/enzymatic	—	Mean comparison	—	High/moderate	[162]
72	Isozyme/phytochemical	NP^9^	Mean comparison	NP	Moderate	[30]
40	ISSR	—	Nei's	0.15–0.9	High	[163]
9	RAPD/ISSR/SSR^5^	UPGMA	Jaccard	0.19–0.73/0.25–0.64/0.26–0.73	High	[164]
32	SSR/STMS^6^	UPGMA	Nei and Li's	0.07–0.79	High	[165]

^1^Number of used accessions, ^2^RAPD: random amplified polymorphic DNA, ^3^AFLP: amplified fragment length polymorphism, ^∗1^H NMR: hydrogen-1 nuclear magnetic resonance, ^4^ISSR: intersimple sequence repeat, ^5^SSR: simple sequence repeat, ^6^STMS: sequence-tagged microsatellites sites, ^7^UPGMA: unweighted pair group method with arithmetic mean, ^8^SAHN: sequential agglomerative hierarchical nonoverlapping, and ^9^NP: not presented in the related reference.

**Table 3:** *Catharanthus roseus* series.

Series	Characteristics
Carpet	Height: 3-4 inches (7.6–10.2 cm), width: 24 in (60 cm), dwarf, great for groundcovers, blooms all summer.

Cobra	Height: 6–12 in (15–30 cm); spacing: 6–9 in (15–22 cm), flower size: 5-6 cm, compact, drought-tolerant, suitable for xeriscaping and growing in container.

Cooler	Height: 10–14 in (30–35 cm), spread: 15–20 cm, low growing compact plants, more tolerant of cool, wet conditions, vibrant colors.

Cora	Height: 14–16 in (35–40 cm), width 22–25 in (55–62 cm), large bold bright flowers, first series to be resistant to the “sudden death,” bloom early, heat tolerant, Disease-resistant to the fungus (*Phytophthora*) causing aerial blight.

Heat Wave	Height: 14–20 inches, remarkable tolerance to heat and drought, blooms early, suitable for containers or hanging baskets.

First Kiss	Height: 10–14 in (28 cm), compact, large-flowered (flower size: 6 cm) and very heavy blooming.

Jaio	Compact, vigorous, heat and drought tolerant, disease-resistant.

Little	Height: 10 to 14 in, best used in small patio planters and decorative pots.

Mediterranean	Grows 5-6 inches tall, cascading habit, fairly vigorous, excellent heat and drought tolerance, performs well in very warm conditions, many color combinations, good for use in hanging baskets or window boxes.

Nirvana	Upright and cascading types, resistant to the fungus causing aerial blight.

Pacifica	Height: 14–20 in (35–50 cm), spread 15–20 cm, open-pollinated group, blooms early, 2-inch flowers with overlapping petals, heat and humidity tolerant, more stress tolerant on the bench and in the garden, very floriferous.

Pretty	Height: 12 inches (30.5 cm), compact, multiflowered plants.

Solar	Heat and humidity tolerant, ideal for early sowings, early flowering, perfect for packs and pots, better disease tolerant.

Stardust	Height: 25–30 cm, compact, many flowered, blooms have a star-shaped, solid white center and bright petal border.

Sunstorm	Large-flowered, compact and tolerant of both hot and cool growing conditions.

Titan	Height: up to 16 in (35–40 cm), spread 10–12 in (25–30 cm), bushy growing habit, vigorous, more disease-resistant than other Vinca series, cool and drought tolerant, earlier flowering, has neat mounding habits, large-flowered, flowers profusely, highest seed quality.

Tropicana	Height 14–20 in, fast-growing, blooms early, large rounded flowers, heat and humidity tolerant.

Tutti Frutti	Large-flowered, heat and drought tolerant.

Victory	Height: 25–35 cm, spread 15–20 cm, intense, clear colors, compact growing habit, disease tolerance, low and early basal branching, formed large round flowers with overlapping petals.

Viper	Height: 20–50 cm, F1 Viper is the king of Vinca-vigorous, large mounded plant covered in massive flowers.

Vitesse	Height: 32–35 cm, compact with basal branching, ideal for early planting.

**Table 4:** Available cultivars of *Catharanthus roseus*.

Series	Cultivars
Carpet	Rose Carpet, Pink Carpet, Magic Carpet.

Cobra	Cobra Apricot, Cobra Orchid W/Eye, Cobra Passion Fruit, Cobra Peppermint, Cobra Purple, Cobra Purple W/Eye, Cobra Red, Cobra Red W/Eye, Cobra Rose, Cobra Strawberry Red, Cobra White.

Cooler	Cooler Apricot; Cooler Blush; Cooler Coconut; Cooler Deep Orchid, Cooler Grape; Cooler Hot Rose, Cooler Icy Pink; Cooler Lavender Halo, Cooler Mixture, Cooler Orchid; Cooler Orchid Deep, Cooler Peppermint; Cooler Peppermint Improved; Cooler Pink; Cooler Raspberry Red; Cooler Red; Cooler Rose; Cooler Strawberry.

Cora	Cora Apricot, Cora Burgundy, Cora Deep Lavender, Cora Lavender, Cora Mix, Cora Pink, Cora Punch, Cora Violet, Cora White, Cora Cascade Cherry, Cora Cascade Lilac, Cora Cascade Polka Dot.

Heat Wave	Heatwave Apricot, Heatwave Blue W/Eye, Heatwave Burgundy, Heatwave Cherry, Heatwave Deep Rose, Heatwave Formula Mixture, Heatwave Grape, Heatwave Midnight Mix, Heatwave Orchid, Heatwave Peach, Heatwave Peppermint, Heatwave Pink, Heatwave Raspberry, Heatwave Red, Heatwave Rose, Heatwave Santa Fe, Heatwave Southwest Mix, Heatwave White.

First Kiss	First Kiss Apricot, First Kiss Blueberry, First Kiss Blush, First Kiss Cherry red, First Kiss Coral, First Kiss Icy Pink, First Kiss Orchid, First Kiss Peach, First Kiss Polka Dot, First Kiss Raspberry, First Kiss Rose, First Kiss Ruby, First Kiss Sunrise, First Kiss Think Pink, First Kiss White.

Jaio	Jaio Dark Red, Jaio Scarlet Eye.

Little	Little Blanche; Little Bright Eye; Little Delicata; Little Linda; Little Pinkie.

Mediterranean	Mediterranean Apricot Broadeye, Mediterranean Cherry Red Halo, Mediterranean Dark Red, Mediterranean Deep Rose; Mediterranean Deep Orchid; Mediterranean Halo Mix, Mediterranean Lilac; Mediterranean Mix, Mediterranean Peach Improved, Mediterranean Peach XP, Mediterranean Pink, Mediterranean Polka Dot; Mediterranean Red, Mediterranean Rose, Mediterranean Rose Red, Mediterranean Rose Hot, Mediterranean White Broadeye; Mediterranean White, Mediterranean Strawberry.

Nirvana	Nirvana Cascade Lavender W/Eye, Nirvana Cascade Orchid, Nirvana Cascade Pink Splash, Nirvana Cascade Rose, Nirvana Cascade Shell Pink, Nirvana Cascade White, Nirvana Pink Blush, Nirvana Red, Nirvana Dark Red.

Pacifica	Pacifica Apricot, Pacifica Blush, Pacifica Burgundy, Pacifica Burgundy Halo, Pacifica Burgundy; Pacifica Cherry Halo, Pacifica Cherry Red; Pacifica Coral; Pacifica Dark Red, Pacifica Deep Orchid; Pacifica Icy Pink; Pacifica Lilac; Pacifica Lipstick Mix, Pacifica Magenta Halo, Pacifica Mix, Pacifica Orchid Halo; Pacifica Peach; Pacifica Pink; Pacifica Polka Dot; Pacifica Punch; Pacifica Punch Halo, Pacifica Raspberry, Pacifica Really Red, Pacifica Red, Pacifica Rose Halo, Pacifica Pure White.

Pretty	Aureo-variegata, Bowles.

Solar	Solar Apple Blossom, Solar Apricot, Solar Blueberry, Solar Blush Pink, Solar Cherry with Eye, Solar Formula Mixture, Solar Fresh Red, Solar Lilac, Solar Orange with Eye, Solar Orchid with Eye, Solar Pink, Solar Raspberry with Eye, Solar Red, Solar Red with Eye, Solar White.

Stardust	Stardust Orchid; Stardust Pink, Stardust Mix, Stardust Rose.

Sunstorm	Sunstorm Apricot; Sunstorm Bright Red; Sunstorm Blush; Sunstorm Lilac; Sunstorm Orchid; Sunstorm Pink; Sunstorm Rose W/Eye; Sunstorm Violet W/Eye; Sunstorm White W/Eye.

Titan	Titan Apricot, Titan Blush, Titan Burgundy, Titan Cotton Candy Mix, Titan Dark Red, Titan Icy Pink, Titan Lavender Blue Halo, Titan Lilac, Titan Mix, Titan Polka Dot, Titan Punch, Titan Pure White, Titan Rose, Titan White.

Tropicana	Tropicana Apricot, Tropicana Blush; Tropicana Bright Eye; Tropicana Pink; Tropicana Rose.

Tutti Frutti	*Agastache*, Rose.

Victory	Victory Apricot, Victory Blue, Victory Bright Eye, Victory Carmine, Victory Carmine Rose, Victory Cranberry, Victory Deep Apricot, Victory Deep Pink, Victory Grape, Victory Lavender, Victory Light Pink, Victory Pure White, Victory Purple, Victory Red.

Viper	Viper Apricot, Viper Grape, Viper Orchid Halo, Viper Pink, Viper Purple, Viper Purple Halo, Viper Red, Viper Red W/Eye, Viper Rose, Viper Watermelon.

Vitesse	Vitesse Apricot, Vitesse Blush, Vitesse Cranberry, Vitesse Fuchsia, Vitesse Grape, Vitesse Hot Pink, Vitesse Lavender, Vitesse Orange, Vitesse Orchid, Vitesse Peach, Vitesse Peppermint, Vitesse Pink, Vitesse Purple, Vitesse Raspberry, Vitesse Red W/Eye, Vitesse Rose, Vitesse Strawberry Red, Vitesse Strawberry Twist, Tropical Orange, Vitesse White.

**Table 5:** List of the most important studies on the medicinal effects of *Catharanthus roseus*.

Application	Modern researches		References
	*In vitro *	*In vivo *	
Acetylcholinesterase and cholinergic antagonism inhibition	Microplate assay	Male Wistar rats^∗^	[166, 167]
Alzheimer's syndrome		Human (clinical trial)	[168]
Anthelminthic activity	*Pheretima posthuma *		[169]
Antiandrogenic activity	Mice		[170]
Antiangiogenesis activity	Chicken eggs		[171]
Antibacterial (antiseptic) activity	Bacteria		[106, 172–175]
Antidysenteric activity	Wistar rats		[176]
Antifertility effect	Male rat		[115, 177, 178]
Antifungal activity	*Trichophyton rubrum* *Hendersonula toruloidea *		[179, 180]
Antihyperglycemic activity (antidiabetic)	Mice, rat, rabbit	Wistar albino rats	[109, 181–188]
Antihypercholesterolemic activity (antihyperlipidemic)	Rabbit, rat		[184, 189]
Anti-inflammatory activity	Rat		[189]
Antimutagenic activity	Micronucleated erythrocytes		[190]
Antineoplastic activity	Mice, rat	Clinical use	[113, 191–198]
Antioxidant activity	Rat		[61, 184, 199]
Antiplasmodial activity	Human erythrocytes		[200, 201]
Antiproliferative activity	Human cells		[202, 203]
Antispermatogenic	Male rat, mice		[204, 205]
Blood cleanser			[206]
Cytochrome P450 inhibition	CYP2D6		[207]
Cytotoxic activity	Human cell line		[208, 209]
Enhances kidney and liver functions	Wistar rat		[185, 210]
Epididymal dysfunction	Rat		[211]
Generate giant spermatogonial cells	Albino rat		[212]
Hypotensive activity	Rat		[108]
Larvicidal activity	*Anopheles stephensi* (malaria vector); *Aedes aegypti *		[213, 214]
Regression of accessory reproductive organs	Male Wistar rats		[215]
Regression of entire reproductive system	Male rat		[216]
Stomachic	—		[108, 217]
Tonic	—		[108, 217]
Tranquilizing and sedative action	—		[101, 108, 217]
Wound healing	Rat		[218]

^*^The research was done *ex vivo*.
